# Complete Genome Sequence of *Salmonella enterica* Serovar Agona Pulsed-Field Type SAGOXB.0066, Cause of a 2008 Pan-European Outbreak

**DOI:** 10.1128/genomeA.01219-13

**Published:** 2014-01-23

**Authors:** Matthew P. McCusker, Karsten Hokamp, James F. Buckley, Patrick G. Wall, Marta Martins, Séamus Fanning

**Affiliations:** a UCD Centre for Food Safety, School of Public Health, Physiotherapy and Population Science, UCD Centre for Molecular Innovation and Drug Discovery, University College Dublin, Belfield, Dublin, Ireland; b Smurfit Institute of Genetics, School of Genetics and Microbiology, Trinity College Dublin, Dublin, Ireland; c Veterinary Food Safety Laboratory, Cork County Council, Inniscarra, County Cork, Ireland

## Abstract

*Salmonella enterica* serovar Agona is in the top 10 most common nontyphoidal serovars reported in humans in the European Union. Here we report the complete genome sequence of an *S. enterica* serovar Agona isolate, designated 24249, that was the cause of a pan-European outbreak in 2008 with 163 confirmed cases reported.

## GENOME ANNOUNCEMENT

*Salmonella* is the second most common bacterial cause of food-borne gastroenteritis worldwide (1). Similarly, in the European Union/European Economic Area (EEA), this organism continues to be the second most commonly reported bacterial cause of gastrointestinal infection after *Campylobacter* but remains the predominant cause of foodborne outbreaks (2). In 2010, a total of 102,323 confirmed salmonellosis cases were reported by the 29 European Union/EEA countries, resulting in an estimated overall burden in the European Union alone of up to 3 billion euros per year (3).

*Salmonella enterica* serovar Agona is an important zoonotic pathogen (4, 5), and in 2010 it became the 10th most frequently reported nontyphoidal *Salmonella* serovar in humans in the European Union, increasing 15% on 2009 (2). It has caused a number of human disease outbreaks in the European Union, as well as internationally, involving a range of foodstuffs, including ready-to-eat savory snacks (6), cereal (7), air-dried raw beef (8), infant milk formula (5, 9), and fennel-aniseed-caraway infusion (10).

In July 2008, the Health Protection Surveillance Centre (HPSC) in Ireland declared an international outbreak in which *S. enterica* serovar Agona was the etiological agent (11). This *S. enterica* serovar Agona strain was designated with a new phage type PT39. Pulsed-field gel electrophoresis (PFGE) performed on isolates recovered from this outbreak showed that they shared the same distinct pattern (designated SAGOXB.0066 by PulseNet Europe). The source of the outbreak was subsequently tracked to an Irish food producer. Some 163 cases in 10 European countries were laboratory confirmed, with ages of those affected ranging from 3 months to 87 years and two elderly patient fatalities. In this study, we sequenced an *S. enterica* serovar Agona strain, designated 24249, that was sourced directly from the food factory at the time of the outbreak.

Whole-genome sequencing of strain 24249 was performed on a 454 GS-FLX titanium sequencer using a combination of shotgun sequencing (GATC, Constance, Germany) and sequencing of a 3-kb mate-pair library (Centre for Genomic Research, University of Liverpool, Liverpool, United Kingdom). An optical map of the 24249 strain was generated to assist genome assembly and subsequent closure (12). *De novo* sequence assembly was carried out using Newbler version 2.6 (Roche), generating 2 scaffolds and 31 gaps. All gaps were closed manually using Sanger DNA sequencing of PCR products. The genome was also submitted to the NCBI Prokaryotic Genomes Automatic Annotation Pipeline.

The *S. enterica* serovar Agona 24249 genome is composed of a 4,762,840-bp chromosome with a GC content of 52.1%. The strain does not contain any plasmids. The chromosome contains 4,543 genes, with 4,342 coding DNA sequences (CDS) identified. There are 91 pseudogenes, 22 rRNA genes (5S, 16S, and 23S), and 80 tRNA genes. Comparison of the 24249 genome sequence to the only other complete *S. enterica* serovar Agona genome sequence (that of SL483) revealed high colinearity. This isolate shows unique genotypic characteristics, including a deletion of a putative phage region.

A detailed report of a full comparative analysis of the genome will be included in a future publication.

### Nucleotide sequence accession number.

The complete genome sequence for *Salmonella enterica* serovar Agona strain 24249 has been deposited at GenBank under the accession number CP006876. The version described in this paper is the ﬁrst version.
